# A Novel Hybrid Multi-Modal Deep Learning for Detecting Hashtag Incongruity on Social Media

**DOI:** 10.3390/s22249870

**Published:** 2022-12-15

**Authors:** Sajad Dadgar, Mehdi Neshat

**Affiliations:** 1 Department of Mathematics and Computer Science, Amirkabir University of Technology, Tehran 15875-4413, Iran; 2 Adjunct Research Fellow at Center for Artificial Intelligence Research and Optimization, Torrens University Australia, Brisbane, QLD 4006, Australia; 3 Faculty of Engineering and Information Technology, University of Technology Sydney, Ultimo, NSW 2007, Australia

**Keywords:** hybrid deep learning models, machine learning models, stacking ensemble, XGBoost, fine–tuning, image–text multimodal classification, object detection, hashtags, social media analysis

## Abstract

Hashtags have been an integral element of social media platforms over the years and are widely used by users to promote, organize and connect users. Despite the intensive use of hashtags, there is no basis for using congruous tags, which causes the creation of many unrelated contents in hashtag searches. The presence of mismatched content in the hashtag creates many problems for individuals and brands. Although several methods have been presented to solve the problem by recommending hashtags based on the users’ interest, the detection and analysis of the characteristics of these repetitive contents with irrelevant hashtags have rarely been addressed. To this end, we propose a novel hybrid deep learning hashtag incongruity detection by fusing visual and textual modality. We fine-tune BERT and ResNet50 pre-trained models to encode textual and visual information to encode textual and visual data simultaneously. We further attempt to show the capability of logo detection and face recognition in discriminating images. To extract faces, we introduce a pipeline that ranks faces based on the number of times they appear on Instagram accounts using face clustering. Moreover, we conduct our analysis and experiments on a dataset of Instagram posts that we collect from hashtags related to brands and celebrities. Unlike the existing works, we analyze these contents from both content and user perspectives and show a significant difference between data. In light of our results, we show that our multimodal model outperforms other models and the effectiveness of object detection in detecting mismatched information.

## 1. Introduction

Over the past decade, with the drastic rise in the popularity of social media, these platforms have played an indispensable role in users’ social lives. They have become more than just a tool for communication and sharing information privately and publicly. Social media platforms provide services allowing users to maintain direct relationships with their followers. Thus, it is a great opportunity for commercial brands and celebrities that encourage them to share visual and textual posts. In other words, social media nowadays can be considered a two-way channel where brands can amplify their marketing strategies, take customer services to the next level and enhance their knowledge about their customers by monitoring activities taking place on social networks [1] and celebrities and public figures can reach a wider audience and monetize with the content they produce. Despite the advantages of services offered to users, some problems may still transpire due to public access to these platforms.

**Figure 1:**
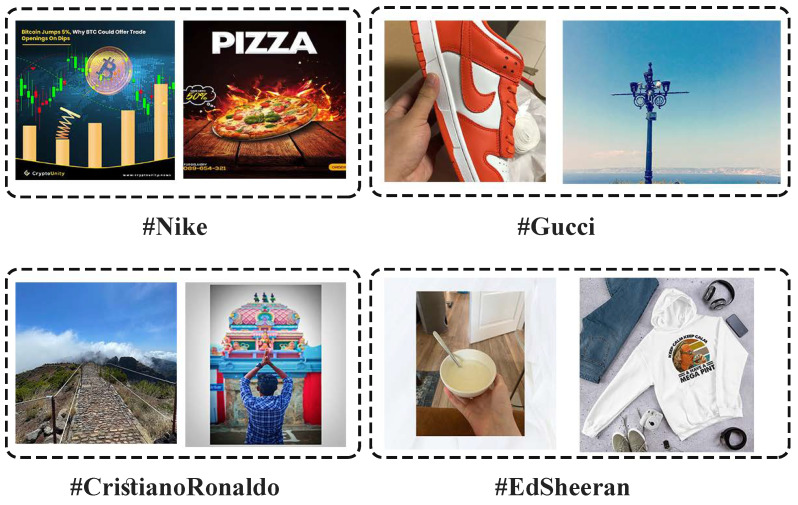
An example of incongruent content that users have shared with irrelevant hashtags on Instagram.

This paper focuses on incongruent Instagram content that can also be referred to as spam, which are posts that do not match users’ expectations [2]. These contents can be found in a variety of cases on Instagram. However, we specifically assess them in hashtag searches. Hashtags are searchable keywords preceded by the hash sign # that are used on social media platforms to categorize information in different contexts. The increase in the number of social searches using hashtags sometimes makes it a substitution for conventional search engines on social media [3]. In addition, by tagging a post, users can contribute and link their posts to other related images and videos. Since using hashtags has no restriction, some users may choose the wrong hashtags inadvertently or intentionally to get more followers or monetize by advertising, which can be annoying for consumers who have a common interest through the hashtag, and also prevent creating a collection of related content that may have valuable insights. Therefore, as illustrated in Figure 1, many irrelevant and incongruent contents on hashtags can be troublesome. Not only are they disturbing and confusing for users searching through the hashtags, but they also impede brands from analyzing their customers in the best way. Additionally, post-hashtag mismatches on Instagram, which are in text and visual formats, have the potential to convey misinformation which is a significant threat [4]. Accordingly, utilizing incongruous hashtags on Instagram posts that comprise both visual and textual data may have adverse consequences. To avoid the potential threats and improve visual-sharing social network performance for any user who spends time on these platforms, there is a need to detect such posts automatically via their visual and textual information.

Computer Vision and Natural Language Processing (NLP) are two fields of Artificial Intelligence (AI) that contain many techniques that can be applied, respectively, to visuals and texts and even combined to solve several real-world problems, such as images with tags on social media [5]. As a result, our primary objective is to find a solution using these techniques that could be used to detect posts with irrelevant hashtags, whether a tag link to an individual or a prominent brand. To this end, we crawl Instagram posts with various information (e.g., images, texts, metadata) from a few hashtags with a large number of contents. In addition, we propose detection models to identify incongruity between posts and hashtags using visual and textual features. Since related objects in images can indicate whether an image is associated with a hashtag or not, we further develop object detection models to detect logos and faces and analyze their performance. Moreover, we analyze the characteristics of mismatched information and users who contribute prevalence of these contents. The key contributions are summarized as follows:

1. We introduce a dataset for Instagram that consists of metadata, visual and textual information collected from different hashtags pertinent to brands and celebrities with additional generic features related to images and texts.
2. We develop machine learning and deep learning models based on metadata, text and images for incongruity detection. We also propose a multimodal model by fusing text and image classifiers. Further, by comparing the experimental results of models, we show that our proposed multimodal models outperform other models.
3. We apply object detection to the two categories of images. First, we use brand-related images to detect a brand’s logos. Second, we employ celebrity-related images to recognize the faces of the celebrity and other people who are somehow connected to them by performing clustering on their Instagram accounts and show the effectiveness of object detection to discriminate incongruent information from other relevant information.
4. We conduct an explorative analysis and empirical study of our dataset from different perspectives to categorize the type of incongruity in posts and examine the characteristic of social media users who share such posts.

The overview of the rest of the paper is as follows. We first review the related literature in Section 2. Then, the proposed approach and our methods are presented in Section 3. We conduct experimental results and explicate data analysis in Section 4. Next, we discuss limitations and provide insight into future works in Section 5. Finally, we conclude and summarize our work in Section 6.

## 2. Related Works

### 2.1. Brand Marketing and Advertising on Social Media

The previous findings reveal that different factors motivate users to use Instagram, including establishing social interaction, recording events and peeking at celebrities [6], who are public figures that have been shown to affect human behaviour strongly [7]. Thus, brands should comprehend these motivations to establish a reinforced relationship with their customers by identifying customer needs and utilizing various advertising techniques that help them obtain a desirable outcome. In recent years, marketers and brands have taken advantage of visual-sharing social networks because visual information is more memorable and provokes stronger emotional reactions than textual information [8]. Consequently, visual brand-related content is a persuasive tool that significantly influences consumers’ buying intentions [9]. Instagram is one of the most popular visual-based social media platforms with a large number of active users. Along with allowing users to share visual information, Instagram, by adding new features in recent years (e.g., product tags on images), created a good marketing atmosphere that can develop more trust between users and companies [10]. Moreover, brands on this platform can implement image strategies that express their concepts and promote the public’s cognitive efficiency [11]. In addition to these benefits, Instagram APIs grant users access to data from business and creator accounts, allowing brands to mine perceptual and semantic features to yield promising results. Many prior studies, therefore, were conducted about marketing and advertising on Instagram. For instance, Liu et al. [12] estimate how brands are represented on Instagram by studying consumer-created images.

In the latest works, employing Machine Learning (ML) and Deep Learning (DL) approaches also allows brands to gain more valuable marketing insight by leveraging disparate information. For instance, Paolanti et al. [13] measured the overall sentiment of brand-related images by proposing a deep Convolutional Neural Network (CNN) model. A Support Vector Machine (SVM) model was presented by Apostolova and Tomuro [14] for extracting named entities in online marketing materials. Wijenayake et al. [15] studied users’ expressions and opinions toward brands and developed a Long Short-Term Memory (LSTM) model to generate and monitor brand personalities. Nakayama and Baier [16] introduced an approach to predict and prevent confusion in a brand’s visual advertisements using CNN. Tous et al. [17] proposed a CNN model to efficiently filter and curate brand-related images from Instagram and Twitter by applying object detection to discover brands’ logos.

In the context of advertising, the privilege of social media in exchanging information at a high level enables brands to create positive attitudes toward their products more than traditional advertising channels. Correspondingly, advertisements have appeared in a variety of forms on social media. Both celebrities and social media influencers who accumulate a high number of followers have the capability to affect a brand’s preferences [18]. One way to advertise, thus, is via exploiting influencers and celebrities, and finding appropriate ones is a challenging task. The most straightforward and relevant task of identifying proper influencers comes from research that suggests a DL algorithm to classify these influencers and disclose the impact of visual congruence on consumers’ brand engagement by analyzing their interaction with their followers [19]. Another way is to customize advertising based on a user’s interests, preferences and personal characteristics to boost engagement [20]. To personalize the advertisement, Hong et al. [21] have also proposed a hybrid interest classification system using Recurrent Neural Network (RNN) and CNN to classify text and images, respectively. Therefore, visual-based platforms are predominantly helpful for brand analysis.

Although social media platforms provide brands with a way to better advertise and exhibit their products that the above works have focused on, some problems can stop brands from completely taking advantage of these platforms. Unlike these works, we attempt to increase the efficiency of hashtag searches in social media, which are crucial for brand marketing and advertising, by identifying incongruent content from congruent content.

### 2.2. Incongruent Content, Misinformation and Spam

Despite the benefits brought by social media to brands, a huge number of unwanted information has been found on these platforms, such as incongruent content [22], spam [23] and misinformation [24] and due to the emergence of new challenges, they have been a top priority in the field of research for the past decade. This content can be broadcasted accidentally or deliberately in a fraction of a second due to the broad audience [25]. Nonetheless, we do not scrutinize the intention of such content, and we examine incongruent content regardless of intent in this study. Incongruence can be defined as information about a specific topic presented in an unrelated context. We investigate a type of incongruent information on Instagram where posts are not associated with their hashtag. Ha et al. [22] worked on the contradiction between brand-related visual data and hashtags by leveraging Computer Vision to analyze and detect these data on Instagram with images, text and meta-data cues. Basically, hashtags are one way to label content and assign it to other related content. When a hashtag is used in a post, the post will emerge on the hashtag’s page. They are beneficial for garnering opinions, surveys and engagement across events. Apart from differentiating between hashtags with visual and textual information, hashtag recommendation methods have also been proven to prevent mismatched information. Alsini et al. [26] reviewed these methods on Twitter and divided them into three categories: first, methods that employ text-based [27], graph-based [28] and classification models [29,30]; next, hybrid user-based methods recommend hashtags based on similarities among users’ interactions and behaviour [31,32]; lastly, hybrid miscellaneous methods whose recommendations are conducted with multimodal features [33]. In contrast to the studies that analyzed irrelevant content, other papers have concentrated on detecting and classifying the images relevant to a company with real-time object detection systems and deep learning techniques on Instagram [34]. Moreover, incongruent content and turmoil and befuddlement caused by the exposure to such information have demonstrated that users were required to make more efforts to process information, which, in the marketing domain, is a negative aspect of a brand [35,36,37].

The discrepancy between information can also be considered an indicator of finding fake news and misinformation [38,39], for example, misinformation detection pertinent to headlines and news [40,41,42]. Misinformation is a type of misleading information that has been disseminated unintentionally and goes through a variety of labels, such as fake news, clickbait and rumors [43]. It is widely accepted that misinformation is a serious menace to societies [44], and the multiple negative impacts of such false information have led researchers to focus on this issue in several areas, ranging from health to marketing. Consequently, previous studies have addressed the task of detecting misinformation, mainly in social media as a source of information. Some have explored the concept from a verbal perspective on text-based platforms such as Twitter, while others presented works from a visual perspective due to the greater deceiving influence [45] and to avoid the dangerous use of social media and technologies that can produce misinformation, such as Deepfake technology [46,47]. Furthermore, with the positive nature of Instagram and the presence of images and video accompanied by textual information, it has provided a place for researchers to work with multimodality by fusing data from several dimensions that have been shown to perform significantly better [48,49]. Amid a wide range of models for detecting misinformation, the dissemination of this content can be amplified by automated fake accounts [50]. In consideration of that, studies were also conducted for Instagram platforms using machine-learning algorithms to discover fake accounts [51,52].

Regarding marketing, brands and companies can also be affected by misinformation in consumer reviews and fake news alongside advertising which undermine consumers’ trust in them and damage brands’ reputations and consumers’ overall attitude toward brands [53]. Among works that assessed this issue, Vidanagama et al. [54] provided a comprehensive analysis of previous research that proposed approaches to detect deceptive consumer reviews. On the other hand, some researchers used consumer reviews for fact-checking. For instance, Zhang et al. [55] developed a model to predict the integrity of answers to consumers’ Question-Answering related to products on Amazon by retrieving evidence from consumer reviews and product descriptions.

The other type of unwanted information that users encounter is spam, which is considered one form of misinformation [56]. Spam is defined as irrelevant and worthless texts and images with a high rate of repetition that is proliferated in any media, like social network platforms and emails. Their form classifies spam into social network spam, image spam, spam links, email spam and advertisement spam [57]. However, allegedly, spam appeared and increased quickly, firstly in emails [58]. The majority of previous work on the processing of spam has been conducted on email text [59], images attached to emails [60,61] and multimodal approaches to eliminate spam from emails [62,63,64]. Even though spam content has become part of the human experience on emails and web, the development of social media platforms and the appearance of spam content brought new challenges to this issue. They lead to a stream of research investigating how to identify and analyze spam on social media. Regarding Instagram, spam content is tied between visual and textual information [65]. Processing of spam content, therefore, is associated with images/videos along with captions and comments. For example, CNN models have been presented with different architectures to detect spam images [2] and spam comments; Complementary Naïve Bayes and SVM models have been developed on balanced and imbalanced datasets of Instagram comments [66]. Other studies focused on extracting texts from spam images by leveraging optical character recognition (OCR), which has shown that its combination with NLP and ML outperforms other ML models trained without OCR [67]. Moreover, the challenges involved in detecting spam content on a large scale have led researchers to look at spam profiles and identify spammers to prevent these accounts from generating spam and remove spam before the user falls for it [68].

Although several methods have been proposed for misinformation and spam, very few works have addressed the problem of incongruent information detection that only focuses on brand-related images. Therefore, we focused on incongruent information detection for brands and celebrities with a large number of followers on social media. Moreover, to the best of our knowledge, very few articles have explored the semantic aspect and the relationship between spam and its context [69]. In this article, we are looking for posts with irrelevant tags in the hashtag search. At first glance and regardless of other posts, a post with irrelevant hashtags might not be spam. However, in the wrong context, when that post is found among other posts that have nothing to do with it, it becomes a worthless post that users do not expect to see among other posts, just like spam.

### 2.3. Machine Learning, NLP and Computer Vision

Promising results over the years obtained by different AI techniques and state-of-the-art methods that have been proposed have led researchers to deal with various problems using these technologies. Although AI technologies are broad and cannot all be mentioned in detail, three areas used in this study should be considered while examining related work; ML, NLP and Computer Vision.

ML is a component of AI that relies on algorithms and data to provide models that are able to perform a specific task automatically with accurate results. Generally speaking, according to the scale of the data and the type of tasks to be performed, different ML algorithms, such as traditional ML and DL, derive benefits from two main learning methods: supervised learning and unsupervised learning. Traditional ML techniques are predominantly supervised learning methods that include a wide variety of algorithms, such as Logistic Regression (LR), SVM, Decision Tree (DT), Naïve Bayes (NB), Random Forest (RF), K-Nearest Neighbor (KNN), etc., each of which has its own advantages [70]. Even though these algorithms are still suited for many tasks independently or as a component of ensemble models, most of them are unsuitable to be used directly in high-dimensional vector information, such as images [71]. Moreover, they cannot work without predefined data. As a result, unsupervised learning methods were performed that allowed models to recognize patterns by themselves and even deal with data that showed up in a matrix form, including images. For example, Zhang [72] proposed unsupervised image clustering algorithms on two datasets to group images into meaningful categories. The drawbacks have also caused traditional ML algorithms to be significantly overshadowed by DL, which can be considered mathematically sophisticated algorithms with a spectrum of architectures capable of solving problems using high-dimensional data [73]. From CNN with its typical layers (e.g., convolution, pooling, fully connected) that are mainly used in image processing and Computer Vision [74] to RNN, LSTM and Gated Recurrent Unit (GRU) with their ability to memorize and recognize sequential patterns in sequential data such as natural language [75]. In addition to these DL algorithms to process these forms of data, it is better to refer to other components of AI that make it possible for computer systems to perceive and process texts and images.

NLP is associated with the capability of a computer system to understand natural language, just as humans, from written or spoken communication, concerning which great strides have been made in performing various tasks such as text classification, sentiment analysis, topic modelling, translation, etc. [76]. Another component of AI is Computer Vision, which is very different to NLP, which includes processing, analyzing and comprehending digital images and videos [5]. In this area, when computer systems’ functions integrate with intelligence, they can fulfil various tasks, such as image classification, object detection, semantic segmentation and so on. Though NLP and Computer Vision are two distinct and active research areas, their combination gives rise to a new interdisciplinary field with an assortment of applications in industries. Some application domains that intersect NLP and Computer Vision include image and video captioning [77], document image classification [78] and visual question answering [79]. Additionally, many studies proposed image–text multimodal and multi-view for classification models that used NLP and Computer Vision techniques to extract textual and visual features [80,81].

A comprehensive summary of the main ML and DL models applied in this field can be found in Table 1.

## 3. Materials and Methods

### 3.1. Approach Overview

In this section, we describe the general approach of our study on incongruent information. The proposed approach to detect this content consists of two primary modules: classification and object detection. This study starts with the classification module, which employed ML and DL methods to identify incongruity between Instagram posts and hashtags. We investigate different models to detect and classify posts based on extracted features from metadata, text and images. We further propose a hybrid multimodal model by fusing image–text classifiers. In the object detection module, we apply an object detection model to brand and celebrity-related images that enables us to identify and recognize related objects from images in our dataset. Accordingly, the first and foremost step is to construct a dataset containing Instagram posts with applicable information that allows us to research the concept of incongruent data.

### 3.2. Data Collection

Before dealing with the main modules, there is a need for a high-quality dataset with multimodal information for the processing to be carried out with the highest success. Hence, we used Instagram, a visual-based social media platform that provides multimodal information. Although there are different types of users on Instagram, the most followed user accounts can be divided into brands and celebrities, which were earlier found to have a higher number of spam comments [82]. Likewise, due to having more visits by other users, it is expected that more incongruent information will be found on hashtags related to prominent brands and celebrities. Therefore, we identified a set of hashtags related to brands/celebrities that have been used frequently in users’ posts and created a dataset of Instagram posts by searching through these hashtags. For retrieving information, we used Instaloader, which is a python library to crawl images and videos along with JSON files containing captions, post engagements (e.g., like count, comment count) and user information (e.g., follower count, post count, profile picture). However, we excluded videos from our study. Instaloader further allows us to garner data based on time intervals. The dataset is aggregated from posts that were published at least 30 days ago to get enough feedback. In total, we were able to gather 12,119 data objects from 8014 users, which we collected from four hashtags.

### 3.3. Data Annotation

Our approach for detecting incongruent information is based on supervised learning that learns from labelled training data. Consequently, the significant challenge is the availability of a dataset with reliable labels, so we annotated the data manually, which is a cumbersome and demanding task. For annotation of our dataset, we created a team of ten people, including seven master’s degree students and three bachelor’s degree students, all with background knowledge of computer science. Based on our experience, finding incongruent information, especially on most-used hashtags, can be identified usually by observation of images and captions. However, detailed discussions were conducted for uniformity of data labelling and acquainting annotators with the task and sample data were provided as a guideline throughout the annotation process. At the beginning of the task, we distributed our dataset among annotators in a way that three annotators evaluated each post. To simplify and accelerate the task, we developed a website that enables annotators to upload their data and evaluate each post by observing textual and visual information. After labelling each post three times, if there was even a single disagreement between the labels, we evaluated them for the last time. According to the obtained results, 76.2% of the data reached a full agreement, and the rest had at least one disagreement, which shows the effectiveness of the guideline and website. Once the data annotation task was completed, we segregated data into “match” and “mismatch” labels. Table 2 presents a list of hashtags used in the dataset with their statistical information regarding the hashtags’ details and distribution of the match and mismatch content in the dataset. According to the table, among 6494 brand-related posts, 39.57% and 60.43% of them were annotated as match and mismatch, respectively. In celebrity-related posts, 38.36% of the samples belong to matched data, and 61.63% belong to mismatched data. As a result, irrelevant hashtags were used in more than half of the collected posts.

### 3.4. Classification Module

This section briefly clarifies the classification module that relies on different ML and DL models with a collaboration of NLP and Computer Vision techniques to address the issue of detecting post-hashtag incongruence. The classification module adopts a four-stage approach. Each stage is explained in detail below:

#### 3.4.1. Metadata Classification

The first stage is carried out by extracting metadata and generic features related to texts and images to quantify the characteristics of posts. Early work in various domains leverages supervised-learning methods that have specifically focused on manually curated features to solve various tasks. To determine the distinctive features between incongruence and congruence information and better analyze their characteristics, we initially explored features that have been analyzed and used for classifying other unwanted information, such as misinformation [83], rumors [84] and spam [85] and also other works on social media that can be seen in Table 1. Then, we selected and mined the most useful features for predictive models and showed their potential to distinguish information in other tasks.

These features are mainly extracted from metadata generated by user engagement and interaction. Instagram generally makes these features available, and we extracted them straightforwardly from the collected data. Besides these features, we also extracted additional features with a bit of computing that indicates a series of attributes related to captions (e.g., word count, sentiment) and images (e.g., size, dominant colors). The overall extracted features from our datasets are listed in Table 3.

Once the features are extracted from raw data, the feature selection takes place to discover the features’ importance. The selected feature vectors representing each post are then fed into different ML and DL classifiers to learn from the metadata features. Analyzing each feature’s characteristic and the classifiers’ performance is discussed further in Section 4.

#### 3.4.2. Text Classification

This stage aims to adopt a DL approach to classify textual information. Text classification is a fundamental task in NLP that has been proven to be solved using DL models by differentiating verbal patterns and distinguishing semantic relations. Here, we developed a text classification model based on a transfer learning approach using the pre-trained Bidirectional Encoder Representations from Transformers (BERT) model to detect incongruence textual information automatically. Textual information generated by the user who publishes an Instagram post can be found in captions, which are titles or brief descriptions of images or videos and also can be found within digital images that refer to text embedded inside of the images. Therefore, our input data are divided into these two corpora. Apart from captions, which are available through the dataset, we need to extract texts from images.

**Figure 2:**
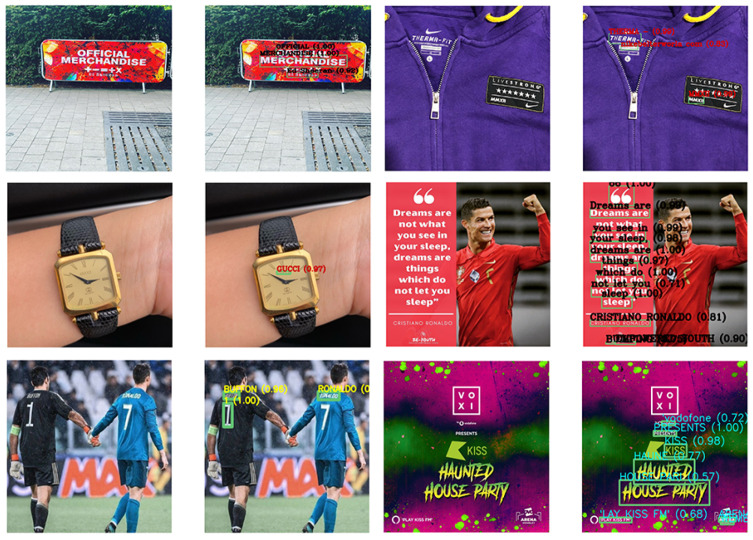
Sample images and recognition of corresponding overlaying text in different hashtags.

Hence, we used OCR to identify and recognize alphanumeric characters automatically and symbols from digital images, including printed, typewritten and handwritten texts, and convert them into a machine-readable text format [86]. With OCR, all words and sentences overlaying the images can be recognized character by character with a confidence rate. Images relate to a scene and texts placed in the background or they are associated with advertising and texts written on objects, as shown in Figure 2. In the dataset, by performing OCR with a confidence rate of 0.7 on the original-sized images, 8159 of the images contained text, and the rest were without any text inside the images. In this study, after preparing and preprocessing the corpus, we fine-tuned the BERT pre-trained model with captions and texts extracted from OCR as two input data.

BERT [87] is a transformer-based model that consists of two steps, i.e., unsupervised pre-training and supervised fine-tuning. The pre-training step was performed on a large amount of unlabeled textual data related to a variety of domains gathered from BooksCorpus and Wikipedia. The fine-tuning step refers to the procedure of retraining the pre-trained model to adapt and perform on a custom dataset. It has been shown that such fine-tuned models outperform traditional models that require large training data sets. In general, BERT serves both as an encoder to process and extract features from input texts and a decoder to employ the features to generate outputs. However, in this study, we used BERT as an encoder to preprocess raw input data and transform them into BERT readable features, i.e., Token IDs, Input Mask and Type IDs that contain 0 or 1, indicating the padding and the token’s sentence, respectively. For encoding, the input data are first tokenized and moved through an embedding layer that can transform each input token into a 768-dimensional vector. Special classification (CLS) and separator (SEP) tokens are then added correspondingly at the first and last of the sentences to clarify each sentence. Finally, positional encoding took place to learn the positions and assign a token to a unique representation based on its context (contextualized embeddings).

#### 3.4.3. Image Classification

Another type of information that can help us to classify our data is images. Images and the features that can be obtained from them give us an advantage that might perform better than the other types of information we mentioned in previous sections. CNN is one of the DL methods that is widely used for image classification. We refer readers to a survey by Rawat and Wang [88] about a comprehensive overview of CNN in the image classification task. Although a wide variety of CNN architectures have been proposed, each depending on the task, Mascarenhas and Agarwal [89] concluded the better performance of Resnet50 on the image classification task by comparing other pre-trained models, including VGG16 and VGG19. Thus, In this stage, we encoded the images using the Resnet50 model, which had been pre-trained on the ImageNet dataset [90] to detect image mismatches.

ResNet [91] is a CNN architecture that enables the construction of networks with thousands of convolutional layers by overcoming the vanishing gradient and exploding gradient problems. In DL models, the deeper the networks get, the less the gradient value changes. Therefore, weights are barely updated during backpropagation. However, Resnet, with its stacked (two-layer) residual blocks that have additional shortcut connections, allows the network to reduce computation and improve performance by skipping some layers and learning with deeper models. Resnet50 is a version of residual networks that consists of 48 convolutional layers along with two pooling layers, i.e., max pooling and average pooling layers. Each two-layer block is replaced with a three-layer block called a bottleneck in ResNet50.

#### 3.4.4. Hybrid Multimodal Deep Learning Model

**Figure 3:**
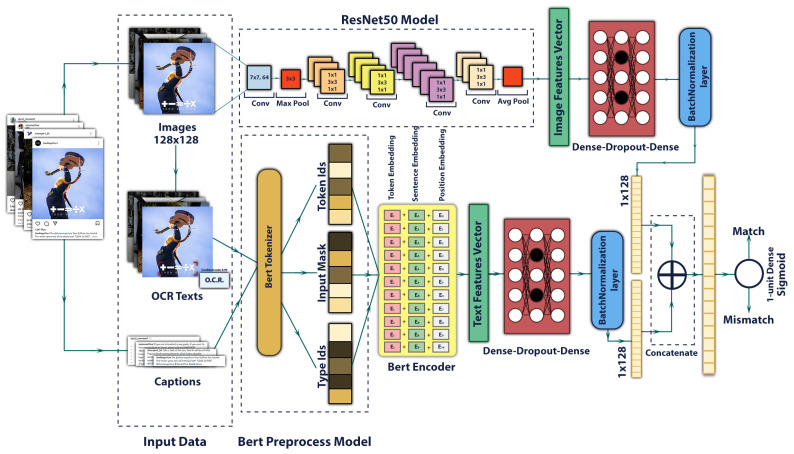
The proposed hybrid multimodal deep learning model.

In real-world problems, it is often the case that the information comes not just from a single modal, but from a multimodal combination of information, just like our tasks. It has been found that multimodal classification can be most effective when text and image diverge semiotically [92]. Therefore, multimodal classification, where image and text are fused, gives us the privilege of strengthening the detection of incongruity information. Until now, we developed text classification and image classification models separately using pre-trained models. In the text classification, we extracted texts from images in the OCR and along with captions, we fed them as inputs to the BERT pre-trained model. We fed input images into a pre-trained Resnet50 model in the image classification. In this stage, however, we proposed a multimodal network by fusing these two models that simultaneously learn from images and textual contents. As illustrated in Figure 3, they moved through the learning process after passing information to the model and extracting embeddings for textual and visual information. The learning process contains fully connected layers accompanied by dropout to reduce overfitting and a batch normalization layer to normalize input features across the batch dimension that improve the training time and add a regularization effect on the network. Finally, we used a late fusion process to concatenate the two modalities and fed them to the final classification layer, a one-unit dense layer with a sigmoid activation function to detect match and mismatch content.

### 3.5. Object Detection Module

In the previous module, we discussed different classifiers which were trained on the dataset. The dependencies of these models on the dataset could be a drawback because content on social media is evolving over time, and the classifiers cannot perform well on the other data as much as on the collected dataset. Nonetheless, relevant posts about a hashtag usually contain the same objects representing the hashtags. Therefore, identifying the objects that appear in most images can effectively detect incongruence information. Note that this module aims to show the ability to detect related objects on images and its performance in identifying incongruence data. As an object detection algorithm, we used YOLO [93], a real-time object detection system that applies a single CNN to the entire image in order to divide regions and predict classes and bounding boxes of the detected object with a probability. Moreover, employing YOLO to detect objects from images in the application has been shown to have advantages in terms of speed and accuracy over other CNN architectures [34]. Therefore, YOLO’s performance in earlier works is another reason for using YOLO in the training process to recognize faces, products and logos.

**Figure 4:**
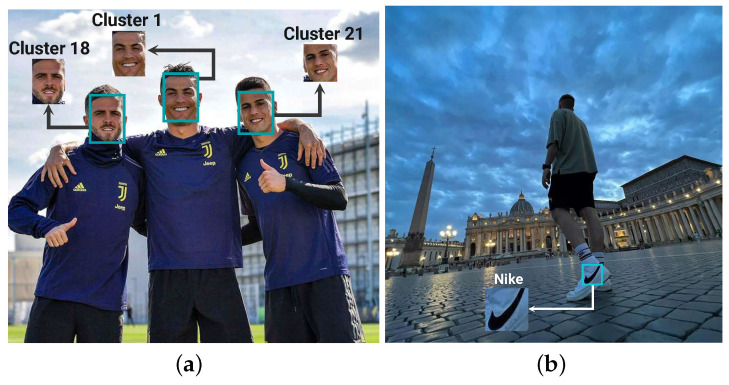
The result of the models in the object detection module. (**a**) face detection, (**b**) logo detection.

As mentioned earlier, we divided the data into two categories: celebrities and brands, which each include different associated objects to detect. For example, faces for celebrities and logos for brands. Thus, this stage is also divided into face recognition and logo detection sections. Figure 4 illustrates the result of face recognition and logo detection.

### 3.6. Face Recognition

**Figure 5:**
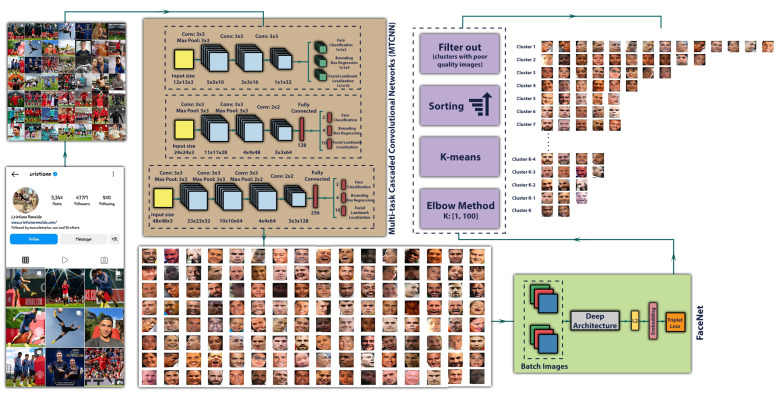
The procedure of face recognition. The images of MTCNN and FaceNet architecture are taken from the corresponding referenced papers.

The most important object, which is an indicator of the presence of celebrities in an image, without any doubt, is their faces because if their faces are present in an image of a post, the post is relevant to a hashtag, and it is right to use the hashtag. The first step for face recognition is to have many images of the target face. Hence, a source of information is required to obtain these images. Instagram is an appropriate place to collect images to recognize celebrities’ faces on Instagram. The Instagram platform lets users share images and save them on their accounts. Therefore, we could find and download all images from their accounts. However, we need to detect and extract the faces from each image before face recognition. We used MTCNN (Multi-task Cascaded Convolutional Networks) [94] as a face detection algorithm containing CNNs in three stages. First, a shallow CNN generates windows and locates candidates. Second, another CNN refines the result of the previous stage and eliminates non-face candidates. Finally, a deep CNN is used to refine the candidates again and returns facial landmark positions such as nose, eyes and mouth. Even though we could extract all faces from the accounts using this algorithm, most faces do not belong to the owner of the Instagram accounts. Consequently, clustering the faces is crucial. To be able to cluster faces, we compacted high-dimensional images of faces into 128-dimensional embeddings using Google’s FaceNet [95], which in light of the face clustering results, has been shown to be invariant to a variety of cases. Finally, we performed K-means to cluster all the obtained faces, and the Elbow method was employed to determine the optimal value of clusters (K). Moreover, we sorted clusters by the number of members and filtered out clusters containing poor-quality images because K-means could not work well due to the quality of images, occlusion, image lighting and persons’ pose in images. Moreover, we eliminated clusters that resulted from a group of faces of different people. The procedure of face clustering is illustrated in Figure 5.

In this way, by performing the steps on Instagram accounts of users who often share pictures of themselves with their followers, we can extract their faces since they appear in a significant number of images. Celebrities are also aware of the effects that social media accounts have on their fans [96] and publish different images of themselves, more than others, to followers. Consequently, the cluster with the highest number of members belongs to the person that owns these accounts. Furthermore, with the right number of clusters that can obtain from the Elbow method, we can also extract other faces from people who are somehow related to the owner of the Instagram accounts. Regardless of removed clusters, the people in images will be ranked based on their appearance on the image in the account by sorting the clusters based on the cluster’s frequency. This means the more a person is present in an image of the respective Instagram account, and the image is placed in higher clusters; thus, the possibility that an image of this person is incongruent with the related hashtag is decreased and vice versa. All faces and corresponding cluster labels were ultimately passed to the training model as inputs.

### 3.7. Logo Detection

The presence of a logo in brand-related images and their detection can effectively differentiate match images from mismatches. The logo is a key component of branding; it is a brand’s emblem, trademark or identity, as it typically joins brand names, products and packaging [97]. As a result, it is a fundamental part of brand-related images shared on Instagram and actually represents the brand. In this section, we used brands’ logos to differentiate mismatched from matched content. Hence, we performed the YOLO object detection model to exhibit the potential of object detection by identifying the logos from images. In the training process, the images of a brand’s logo were first collected via the brand’s Instagram account and Google Images, containing icons and images of logos overlaying on the products. Then, the logos were labelled using LabelImg, a tool for labelling bounding boxes in images. After the labelling process, the images and their labelled bounding box passed through the training model to learn objects from images.

## 4. Experiment and Analysis

### 4.1. Data Analysis

In this section, we discuss an extensive analysis of incongruent information and explicate the characteristics of incongruent information and users that use irrelevant tags.

#### 4.1.1. Mismatch Topics

We conducted an empirical study to discover the relation among the hashtags and their various irrelevant topics and attempt to find answers to different questions, which can help us to gain a better comprehension of this content. For example, which topics include incongruent information, which topic constitutes the majority of this information and so on. As mentioned in the discussion on data collection, we collected data for two hashtag categories for which consumers post many photos: brands and celebrities. We found incongruent hashtag-post information in 60.42% of brand data and 61.63% of celebrity data and categorized them into different topics based on observation. Although there are several ways to categorize mismatched information based on their topic, generally, the major topics on Instagram were:

- Personal: selfies and photos of an individual or group without any relation to the hashtag.
- Art: painting, graphic art, musical instruments and artists.
- Sport: sports equipment, pictures of professional sports, athletes.
- Animal: all pictures of animals.
- Food: meals and beverages and simply everything edible and drinkable.
- Cosmetic: hairdressing, makeup, cosmetic treatments, even healthcare.
- Environment: photo of nature, building.
- Quote: images of quotes, memes, tweets, manuscripts.
- Screenshot: photos displayed on the screen of a computer or mobile phone.
- Ads: posters and flyers.
- Economy: images relating to bitcoin and other digital currencies.
- Shop: online sales and products related to other brands.
- Inappropriate: sensitive and sexual pictures that are not suitable for all users. Note that Instagram strictly handles this sort of content, so there is little of them.
- Other: the remaining images do not belong to mentioned topics.

**Figure 6:**
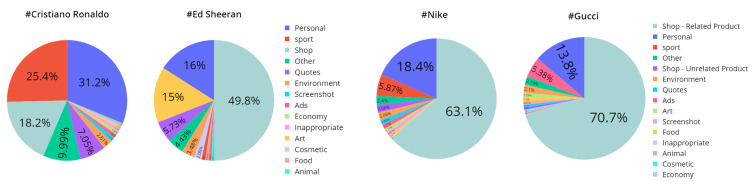
Distribution of the main mismatch topics over posts in the dataset.

In addition, we categorized the shop category in the brand-related hashtag into related and unrelated products due to the different nature of these two types of hashtags. Figure 6 shows the proportion of topics for each hashtag in the dataset. As illustrated in Figure 6, in both brands’ hashtags, the most common type of incongruent information involves brand-related products from other brands and more than half of the posts belong to this topic. As a result, it makes it difficult for our classification models to distinguish between brand-related data. On the other hand, in celebrity-related mismatches, the theme of some data is similar to the corresponding hashtag. For example, in #CristianoRonaldo, which is associated with an athlete, the sports topic has a high percentage. In #EdSheeran, which is related to an artist, the art topic forms a large part of the data. Moreover, it can be inferred that online shops and users who sell products on Instagram use celebrities’ tags to attract users who constantly or occasionally visit these hashtags since a significant part of incongruent information in celebrity-related hashtags comprises the shop topic. In addition, the role of personal content is undeniable, which intertwines with mismatches in all hashtags.

#### 4.1.2. Hashtag Analysis

**Figure 7:**
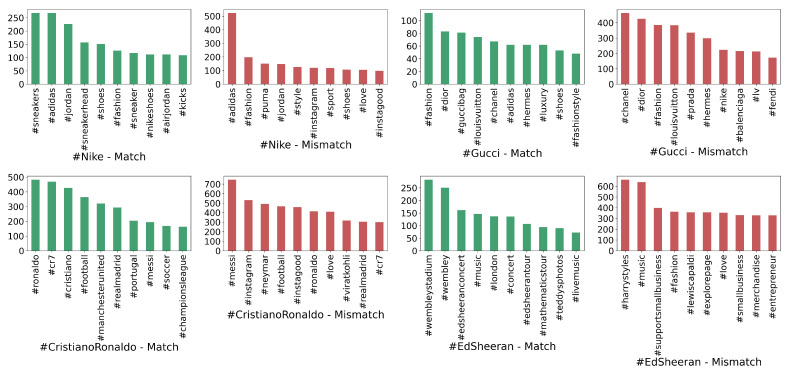
Frequency of the top 10 hashtags about the match and mismatch content in each category on Instagram.

In Figure 7, by ignoring the target hashtags that have been used for data collection and consequently are present in all sample data, we listed the most frequent hashtags in each tag used in the dataset. We found differences between the hashtag distributions in match and mismatch content. While in the congruent content, hashtags mainly refer to the target hashtag’s general idea, those in the incongruent content are pertinent to diverse themes with much more repetition throughout all Instagram posts. For instance, in #EdSheeran, the congruent information is about music, concerts and tours. However, the incongruent information includes hashtags about other artists, fashion, love and business. As a result, we conclude that other unrelated hashtags that have nothing to do with each other can be found frequently in this type of content.

**Figure 8:**
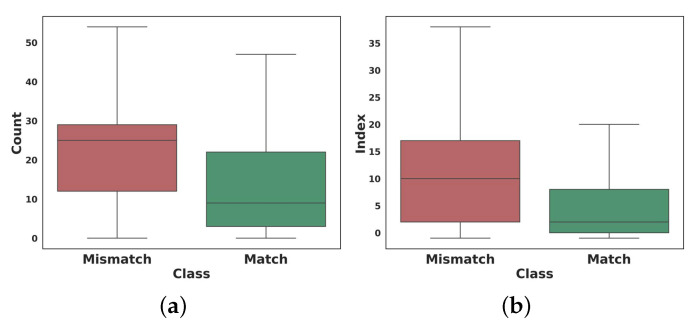
The comparison of hashtags in matched and mismatched content (the outliers are ignored), (**a**) the number of hashtags, (**b**) the Hashtag sequence index.

Another analysis that can be pointed out about hashtags is the number of hashtags and the order of their placement in a single caption. As illustrated in Figure 8a, we discovered a significant difference between the number of hashtags in a single post. Users sharing incongruent content tend to use more hashtags in their posts to be seen by more visitors. Furthermore, considering the hashtags of a post as a sequence of tags, we obtained the index of the target hashtag in the sequence in each sample datum. As shown in Figure 8b, the target hashtag usually appears in the congruent information at the beginning of this sequence. In comparison, the hashtag in incongruent information may occur at the end of the sequence.

#### 4.1.3. User Analysis

**Figure 9:**
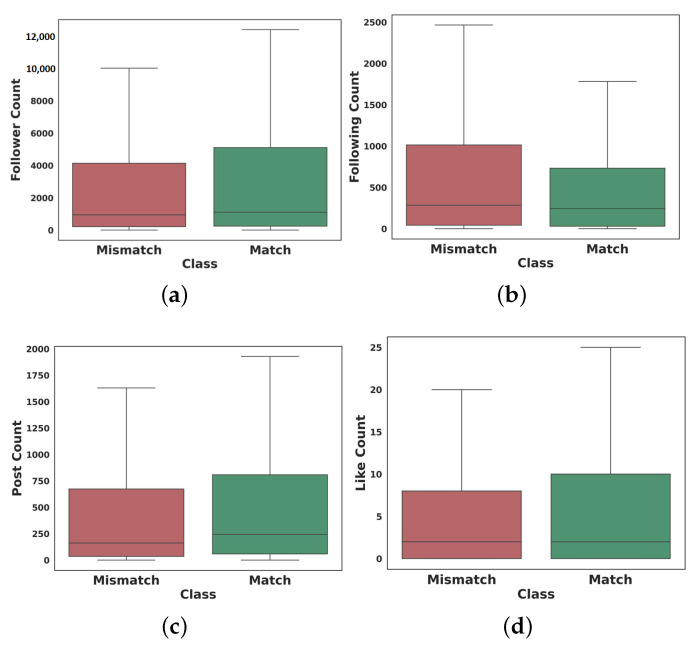
User engagements in the match and mismatch content (the outliers are ignored), (**a**) Follower count, (**b**) Following count, (**c**) Post count, and (**d**) Like count.

Other than content analysis, we examine the characteristics of users who are responsible for creating mismatched information by using irrelevant hashtags. As mentioned in the data collection discussion, the data were collected at least a month after their being published on Instagram. So, enough feedback and reactions had been received from others. As shown in Figure 9, based on the user engagement information obtained by measuring the audience’s interaction in the sample posts, users who shared incongruent information followed more users and were followed less by others. Moreover, the number of posts of these users is less than users who create congruent information. In addition, although congruent posts received more likes, there was no significant difference in the number of comments.

**Figure 10:**
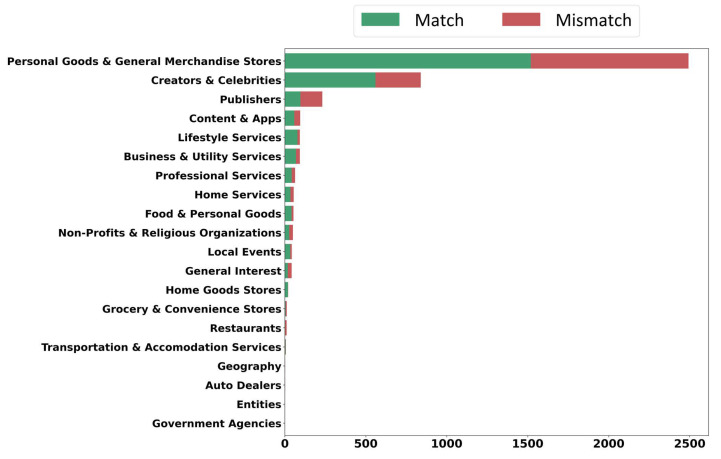
The comparison of business accounts in the dataset.

Moreover, Instagram enables users to create business accounts that provide additional features that help them to expand their business and improve their strategies, such as the ability to run advertising, access to insights to analyze their profile, posts and more. Moreover, as part of the accounts set up for business, Instagram allows users to select a business category from hundreds of categories, letting visitors understand their type of business better. In our dataset, business accounts comprise 34.92% of the total data in 20 categories. Among these categories, “Personal Goods & General Merchandise Stores” is the most common category, followed by “Creators & Celebrities” and “ Publishers”. According to Figure 10, each category has more congruent information, which shows that users who do not have business accounts are more involved in generating incongruent information.

**Figure 11:**
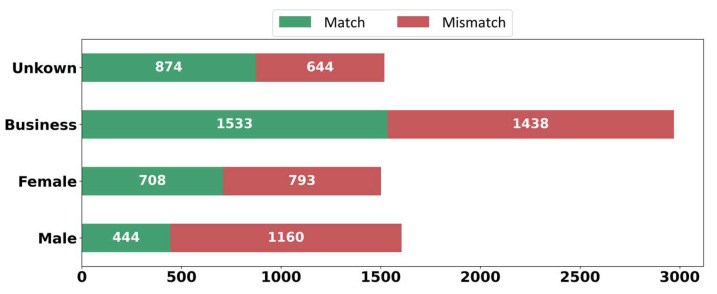
Comparison of the gender of users.

Finally, we investigated gender to fulfil our analysis of users. Since such information cannot be obtained from Instagram APIs, we carried out another empirical study to examine users’ gender. At the time of the data collection process, we stored the profile image of each user along with other features. In this study, we divided the users into four groups based on their profile pictures: business, male, female and unknown. The unknown group includes profile pictures concerning which the gender cannot be determined due to not setting a profile picture or using fake pictures. Moreover, due to the limitation under the API, we could not extract some profile URLs in JSON files. From Figure 11, we discover that the most frequent group belongs to businesses and males share a slightly higher percentage of mismatched content than females.

### 4.2. Experimental Results

In this section, we discuss the experimental setup and the performance evaluation of the classification and object detection models.

#### 4.2.1. Feature Selection

**Figure 12:**
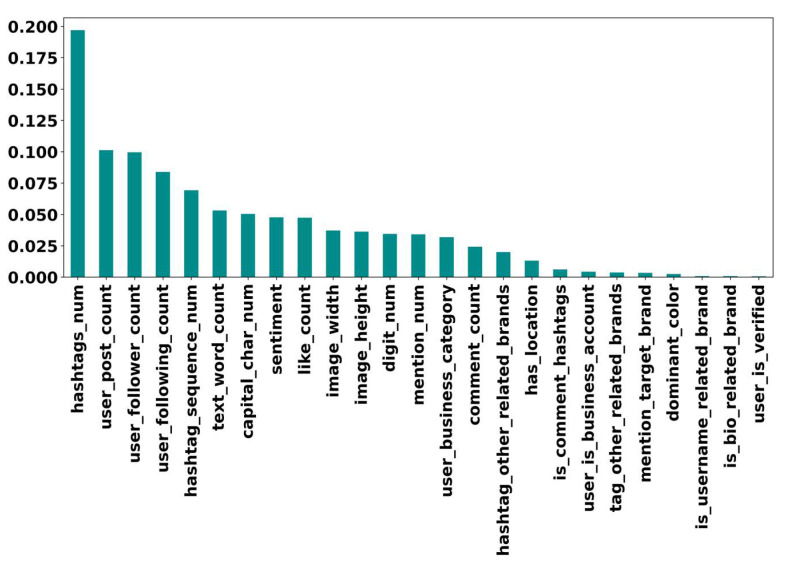
The feature importance of the collected features using Random Forest.

In Section 3.4.1, we extracted features from the collected dataset. To discover those features that contribute most to differentiating incongruent information from congruent information and exclude redundant and irrelevant variables from the model training, we performed Recursive Feature Elimination (RFE). RFE is a recursive feature selection algorithm that identifies the important features based on an estimator’s accuracy. In this experiment, we used RF as the estimator of RFE. Moreover, we performed feature importance via RF to better demonstrate the impact of the extracted features and their discriminative power. As shown in Figure 12, the number of hashtags that were also analyzed in Section 4.1.2 has the most impact on classification. In the following, user engagement features are in the next ranks. In contrast, due to the limited number of samples with corresponding characteristics, most features were extracted from the captions and other features (e.g., user_is_verified) have less contribution.

#### 4.2.2. Classification Results

In the classification module, the collected dataset was initially divided into training, test and validation sets with an 80:10:10 ratio. We tested different architectures and tuned the hyperparameters to find the optimal models. All hyperparameters used for each method are enumerated in Appendix A. Then, the models were built using the collected dataset, as described in Section 3. In metadata classification, after performing feature selection, we used ML algorithms, including SVM [98], Stacking Ensemble ML [99], RF [100], XGBoost [101] and Deep Dense layers to train classification models. In addition to the pre-trained models described in Section 3, we also used these ML models for text and image classification to provide a comprehensive experiment. In the text and image classification tasks using these algorithms, the encoded data are obtained from the pre-trained models and used as the inputs of the ML models. The learning process was conducted using scikit-learn and TensorFlow to build the BERT, Resnet50 and VGG19 models and other ML models. The pre-trained models were fine-tuned on the collected dataset. Then, the Adam optimizer was used during the model training with a learning rate of 0.001 and a batch size of 32 for 100 epochs. The model with the best validation performance was used for evaluation. To evaluate the performance of the models in the classification module, we used accuracy and F-score, which are shown in Table 4 and Table 5, respectively.

(1) $$F\text{-}\mathrm{score}=\frac{2\times \mathrm{Precision}\times \mathrm{Recall}}{\mathrm{Precision}+\mathrm{Recall}}$$

(2) $$\mathrm{Precision}=\frac{\mathrm{TP}}{\mathrm{TP}+\mathrm{FP}}$$

(3) $$\mathrm{Recall}=\frac{\mathrm{TP}}{\mathrm{TP}+\mathrm{FN}}$$

(4) $$\mathrm{Accuracy}=\frac{\mathrm{TP}+\mathrm{TN}}{\mathrm{TP}+\mathrm{TN}+\mathrm{FP}+\mathrm{FN}}$$

Based on the results obtained from the models, the image–text multimodal architecture, as expected, obtained relatively more satisfactory results than text and image classification separately. Moreover, the results indicate that integrating caption with OCR texts yields better results than classification without OCR in most cases. In addition, we observe a slight difference between the performance of the two types of hashtags in some classifiers. These models cannot classify data in brand-related hashtags as much as in celebrity-related hashtags. Our empirical study on the type of mismatched content can justify these differences. As shown in Figure 6, there was a large volume of incongruent information about brand-related products from other brands, which makes it difficult for our models to discriminate between them. Therefore, the object detection model can help in this case due to its ability to detect logos, among other related product images. Moreover, as stated in Section 3, it has been shown that the Resnet50 model outperforms other pre-trained models, such as VGG19. However, we again tested VGG19 for the image classification task and compared it with Resnet50. As a result, Resnet50 is employed in the multimodal model.

#### 4.2.3. Object Detection Results

Additional experiments were performed by focusing on the object detection module. In the second module, we used additional images and fed them into YOLO to detect faces and logos from images in the collected dataset. First, to recognize faces from posts in celebrity-related hashtags (#CristianoRonaldo and #EdSheeran), we downloaded all images from their account (@cristiano and @teddysphotos) and performed the procedure which contains face detection, finding the optimal number of clusters using the Elbow method, clustering the faces with K-means and filtering out clusters with low-quality of faces and clusters with faces belonging to different people (unknown). The statistics of face clustering are shown in Table 6. Afterwards, the related faces with their cluster label were fed as the input to the model. Second, to detect brands’ logos, we downloaded 1000 images of each logo, including logos overlayed on products and their icons from the corresponding Instagram accounts and Google Images. Ultimately, the images and their labelled logo bounding boxes pass through to the object detection model.

In the learning process, we split the additional visual data into training and test sets with an 80:20 ratio and ran the YOLO model with a batch size of 64 over 1000 epochs. To measure the performance of the YOLO models, which make predictions in terms of bounding boxes and labels, we used Mean Average Precision (mAP). the mAP is obtained from the average of AP, which is calculated by averaging the precision of recall values for each class.

(5) $$\;\mathrm{mAP}=\frac{1}{n}\sum_{k=1}^{k=n}\;{\mathrm{AP}}_{k}=1,\;{\mathrm{AP}}_{k}=\mathrm{The}\mathrm{AP}\mathrm{of}\mathrm{class}k,\;\;n=\mathrm{the}\mathrm{number}\mathrm{of}\mathrm{classes}$$

**Figure 13:**
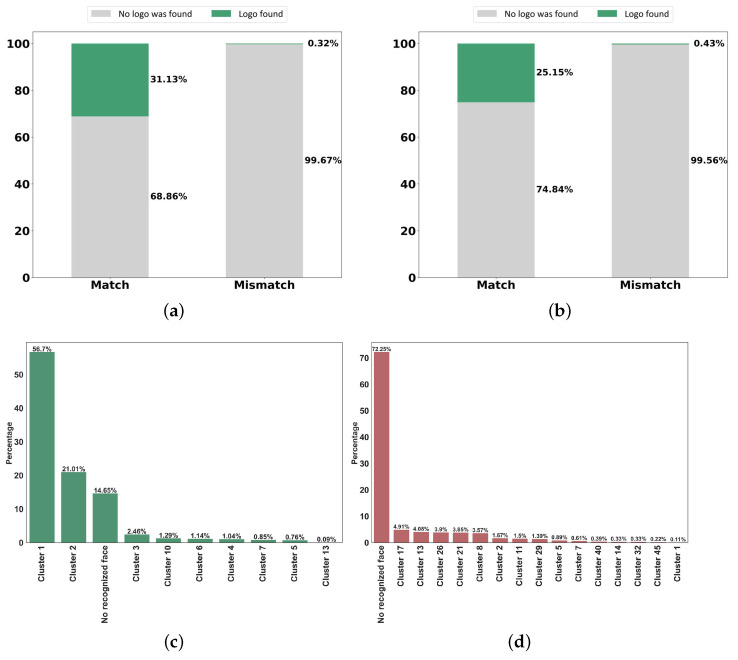

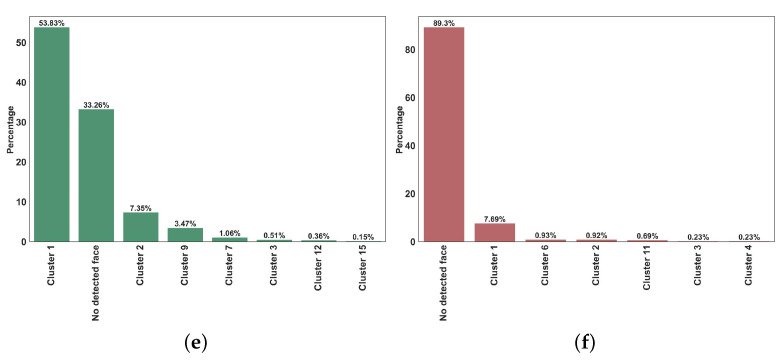
The result of YOLO on the dataset. (**a**) Logo detection on the #Nike hashtag. (**b**) Logo detection on the #Gucci hashtag. (**c**) Face recognition on the #CristianoRonaldo hashtag with a Match label. (**d**) Face recognition on the #CristianoRonaldo hashtag with a Mismatch label. (**e**) Face recognition on the #EdSheeran hashtag with Match label. (**f**) Face recognition on the #EdSheeran hashtag with Mismatch label.

Based on our experiments, for models trained on brand-related images, the mAP values are 0.84 and 0.81 for images pertinent to Nike and Gucci, respectively. For celebrity-related images and recognizing their faces, since each face is extracted from images and the bounding box is set to the size of the image, the mAP is not a good metric to evaluate the model. We used the accuracy to measure what percentage of faces could be detected by their correct cluster labels. The result obtained by performing the model on the test datum is 87.43% for CristianoRonaldo and 72.61% for EdSheeran. Finally, to demonstrate the ability of object detection models, we perform the models on the visual data in the collected dataset. In light of the results, Figure 13 illustrates the logos and faces detected by the object detection model on the match and mismatch data in each hashtag. Based on the result obtained from running the trained models on the images of hashtags, we noticed that models were able to recognize faces and logos in many matched images. Nonetheless, it is expected that a better result will be obtained by using more data in the training process.

## 5. Limitations and Future Works

This research explored hashtags pertinent to brands and celebrities to identify and filter out incongruent information. Hashtags also play an essential role among people during critical situations; for example, #COVID-19, is used worldwide for notifying people about the pandemic and other hashtags have helped people to be informed about occurrences and events by using them frequently and becoming a trend. Therefore, future work can concentrate on these topics as a source of information, which contain a high amount of unwanted information. This study also has several limitations that need to be explored in future research. First, we have explored incongruent information regardless of videos. Future work can investigate videos by developing methods that can be applied to audio and video. Second, we extracted several features to analyze and classify data based on content and user characteristics. Nonetheless, more features can still be extracted from text and images in the future. Third, we employed grid search to optimize the hyperparameters in the models. In addition to grid search techniques, many methods could be addressed. Future works could address the application of optimization methods to adjust the hyper-parameters and develop faster and more effective auto-tuners, such as methods used in [102,103]. Fourth, as mentioned in Section 4, our classification models depend on the dataset, which brings some limitations. Other than object detection models used in this paper, future studies can focus on real-time methods to overcome these limitations. Finally, although some papers have conducted experiments to investigate the performance of object detection models in terms of speed and consistency, the same as [34], some experiments could still be conducted by applying different object detection models to detect related objects in this task.

## 6. Conclusions

In this research, we presented work on post-hashtag incongruent information and discussed their prevalence in hashtags searches of brands and celebrities. We initially collected a dataset consisting of Instagram posts and annotated it into match and mismatch labels. Then, we conducted our research in two modules: classification and object detection. In the classification module, we proposed methods that adopt DL, NLP and Computer Vision to detect incongruent contents from different aspects, including metadata, text and image. We also proposed a hybrid multimodal DL model based on transfer learning to learn simultaneously from visual and textual information. In the second module, to illustrate the ability of object detection models to discriminate between matched and mismatched information, we performed YOLO on the images in the dataset to recognize faces and logos related to the hashtag. For face recognition, we trained the model using faces extracted from Instagram using a novel pipeline that ranks the faces based on the number of their appearances on an Instagram account. We also trained the logo detection model using images of logos collected from the brands’ Instagram accounts and Google Images. To demonstrate the potential of our approaches in the two modules and analyze the data, we conducted experiments on the dataset. In particular, the results indicate that leveraging from both image and text simultaneously improves the results compared to other models. Furthermore, the results suggest that detecting related objects, which are the identities that link the posts to the hashtag, particularly helps to differentiate between matched and mismatched information. Finally, we conducted an explorative analysis and empirical study on our dataset. In the data analysis, we investigated characteristics of incongruent content and discussed the differences between topics, hashtags, engagements and user accounts.

## Figures and Tables

**Table 1:** A summary and comparison of the main characteristics of related ML models in the literature.

Ref.	Year	Task	Source of Information	Dataset	Data Type	Models	Main Focus
[22]	2020	Mismatch Detection	Instagram	7769 labeled posts and 444,491 unlabeled posts	Textual, Visual, Metadata	LR, SVM, RF	Detecting brand-irrelevant posts in brand-relevant hashtags
[40]	2020	News-Headlines Incongruent Detection	News	1.7 million news articles	Textual	SVM, DL	Introducing a web interface for predicting the incongruence between news-headline
[41]	2022	News-Headlines Incongruent Detection	News	Incongruent News Headline Dataset	Textual	Deep Learning (GRU)	Proposing a method to detect incongruence of news headlines using the lexical and contextual connection between news body and its headline
[42]	2020	News Headlines Incongruent Detection	News	NELA17: 91,042 news, Clickbait Challenge: 21,033 social media posts	Textual	SVM, LSTM	Incongruence detection using inter-mutual attention-based semantic matching
[27]	2021	Hashtag Recommendation	Twitter	30 million news tweets	Textual	SVM	Proposing a novel method to recommend hashtags of tweets using lexical, topical, semantic and user influence features
[29]	2020	Hashtag Recommendation	-	Two public datasets: 18,464 articles with five tags and 127,600 articles with four tags	Textual	AdaBoost, RF, LSTM, Bi-LSTM, CNN	An approach to recommend hashtags using text classification.
[30]	2020	Hashtag Recommendation	Instagram	HARRISON dataset: 57,383 multi-labeled images	Visual	Voting Deep Neural Network with Associative Rules Mining	Recommending one to ten hashtags for images
[31]	2016	Hashtag Recommendation	Twitter	1,674,789 tweets with 28,526 hashtags	Textual	Latent Dirichlet Allocation (LDA)	Hashtag recommendation using the latent relationship between words and hashtags
[32]	2020	Hashtag Recommendation	Twitter	Dataset-UDI-TwitterCrawl-Aug2012	Textual	Clique percolation method (CPM)	A community-based approach to recommend hashtags using tweet similarity
[33]	2022	Hashtag Recommendation	Social media	-	Textual, Visual, User information	Hybrid deep neural network	Proposing a multimodal personalized hashtag recommendation
[49]	2020	Misinformation Detection	Instagram	30,000 posts	Textual, Visual	LSTM, GRU, VGG16, VGG19, ResNet50, ResNet101, DenseNet121, DenseNet169, Ensemble model	Detecting medical misinformation with semantic level and task-level attention to focus on important contents
[39]	2020	Fake News Detection	-	George McIntires dataset: 10,558 texts	Textual	LSTM, FNN	Fake news detection using NLP and deep learning by including auxiliary features from live data mining
[51]	2020	Fake account Detection	Instagram	10,000 accounts	Metadata	SVM, RF, NB, DT, MLP	Introducing a method to identify fake accounts efficiently
[52]	2019	Fake Account Detection	Instagram	Two datasets: 1002 real and 201 fake accounts; 700 real and 700 automated accounts	Metadata	NB, LR, SVM, NN	Detecting fake and automated accounts
[55]	2020	Fact-Checking	Product Q&A forums on Amazon	60,864 answer claims about products	Textual	CNN, LSTM, AVER (proposed model)	Proposing AVER, a model to predict the veracity of answers based on evidence
[25]	2019	Spam Detection	Twitter	2000 Dialectical Arabic tweets	Textual	SVM, NB	Detecting malicious and spam content on Twitter written in Dialectical Arabic
[59]	2019	Spam Detection	Email	962 emails	Textual	NB, SVM	Effect of preprocessing of text on the performance of models
[60]	2016	Spam Detection	Email	52,934 images in 7 categories	Visual	CNN and SVM instead of the Softmax layer	Classifying spam images into seven categories
[61]	2022	Spam Detection	Email	1,725,928 spam images extracted from real spam emails	Visual	RF, DT, KNN, SVM, NB, CNN	Classifying spam images and analyzing the performance of ML models
[62]	2008	Spam Detection	Email	14,723 emails	Textual, Visual	DT	Proposing a system to filter out spam emails using different sets of features
[63]	2019	Spam Detection	Email	Text dataset: 2893 message, Image dataset: 2359 images	Textual, Visual	SVM	Propose a method to improve spam classification using a dataset with a small number of data
[64]	2017	Spam Detection	Email	1251 spam images from emails, Enron Spam Dataset: 33,645 texts	Textual, Visual	CNN	Detecting spam emails with hybrid architecture
[2]	2019	Spam Detection	Instagram	8000 images	Visual	CNN	Detecting spam images and comparing five different CNN architectures
[66]	2019	Spam Detection	Instagram	2600 comments	Textual	SVM, Complementary NB	Detecting spam using a balanced and an imbalanced dataset
[67]	2020	Spam Detection	-	Mark Dredze spam images: 10,000 images	Textual, Visual	DL	Extracting text from images using OCR to improve spam classification
[68]	2021	Spam Profile Detection	Instagram	916 user profiles	Metadata	MLP, RF, KNN, SVM	Detecting spammers by extracting additional features
[12]	2020	Image Classification	Flickr, Instagram	13 features about color, shape, and texture from 16,368 images	Visual	SVM, CNN	Measuring how brands are portrayed on social media
[19]	2020	Image Classification	Instagram	More than 45,000 images	Visual	CNN	Classifying images’ themes and analyzing to reveal the hidden relationship between visual content and brand engagement
[17]	2018	Image Recognition, Object Detection	Instagram, Twitter	More than 50,000 images in 100 categories	Visual	CNN	Minimizing manual curation of brand-related images
[34]	2021	Image Recognition, Object Detection	Instagram	Starbucks Instagram images	Visual	Mask R-CNN, Faster R-CNN, YOLO, SSD	Proposing a model to recognize the identity of a brand using object detection
[72]	2021	Image Clustering	Chinese social media, Instagram	Images of protests, images related to climate change	Visual	K-means, Deep-Cluster	Developing three image clustering algorithms on two datasets
[13]	2017	Sentiment Analysis	Instagram	GfK Verein Dataset: 4200 positive, negative and neutral images	Textual, Visual	Deep CNN, KNN, SVM, DT, RF, NB, ANN	Estimate the overall sentiment of brand-related pictures from social media.
[21]	2020	User Interest Classification	Instagram, Twitter, Facebook, Flickr, Google	33,647 images and 21,022 texts	Textual, Visual	CNN, RNN	Improving personalized advertising based on users’ interests
[14]	2014	Named Entity Recognition (NER)	Online sources	1920 online flyers	Textual, Visual	SVM	Recognizing 12 types of named entities in online marketing materials
[16]	2020	Predicting Brand Confusion	All channels	Image and video advertising	Visual	CNN	Proposed an approach to predict the uniqueness of brand positionings
[15]	2021	Generate Brand Personalities	Social media data	1.2 million posts	Textual	Deep LSTM	Investigating how users’ opinions can be used to generate and monitor brand personalities

**Table 2:** The distribution of the collected data with additional statistical information for each hashtag.

Type	Hashtag	Total Number of Posts	No. of Collected Posts	No. of Matches	No. of Mismatches	No. of Users
Brands	#Nike	125.6 million	3151	1531	1620	2266
	#Gucci	69.4 million	3343	1039	2304	1940
Celebrities	#CristianoRonaldo	12.7 million	3481	1024	2457	2405
	#EdSheeran	5.6 million	2144	1134	1010	1403

**Table 3:** List of extracted features with their descriptions.

Type	Feature	Description
User	user_follower_count	Number of followers
	user_following_count	Number of followings
	user_post_count	Number of posts published by the user
	user_business_category	Type of accounts business
	user_is_business_account	Type of the account
	user_is_verified	Whether the user is verified by Instagram
Post	like_count	Number of the post’s like
	comment_count	Number of comments
	has_location	Whether the location has been specified by the user in the post
	mention_num	Number of mentions (@)
	hashtags_num	Number of hashtags (#)
Text	sentiment	Sentiment of captions
	text_word_count	Number of words in captions
	capital_char_num	Number of capital characters
	digit_num	Number of digits
	hashtag_sequence_num	Index of a target hashtag in a sequence of hashtags
	is_comment_hashtags	Whether hashtags are used in the caption or comments
	mention_target_account	Whether the user also mentions the target account
	hashtag_other_related_brands	Whether the user tagged other famous brands
	tag_other_related_brands	Whether the user mentions other famous brands
	is_bio_related_brand	Whether the bio of the account relates to the target account
	is_username_related_brand	Whether the username relates to the target account
Image	dominant_color	The dominant color of the image
	image_original_size	Size of the image

**Table 4:** The accuracy of the models on the test set. The best-performed model shows in bold.

Type	Models	#Nike	#Gucci	#CristianoRonaldo	#EdSheeran	All Hashtags
Metadata Classification	SVM	0.6518	0.6956	0.7106	0.7558	0.7112
	Stacking Ensemble	0.6518	0.6895	0.7220	0.7883	0.7194
	RF	0.6993	0.7492	0.7841	0.7868	0.7568
	XGBoost	0.7267	0.7522	0.7965	0.7930	0.7582
	Deep Dense layers	0.6990	0.7019	0.7177	0.7341	0.7417
Text Classification (Without OCR)	SVM	0.6307	0.6542	0.6760	0.6832	-
	Stacking Ensemble	0.6174	0.6412	0.6497	0.7037	-
	RF	0.6234	0.6955	0.6607	0.7204	-
	XGBoost	0.6429	0.7004	0.6946	0.6981	-
	Fine-tuned BERT	0.7358	0.7448	0.8142	0.8096	-
Text Classification (With OCR)	SVM	0.6317	0.6483	0.6814	0.6722	-
	Stacking Ensemble	0.6192	0.6559	0.6507	0.7305	-
	RF	0.6344	0.6784	0.6637	0.7253	-
	XGBoost	0.6830	0.7067	0.6911	0.7629	-
	Fine-tuned BERT	0.7509	0.7640	0.8172	0.8342	-
Image Classification	SVM	0.5889	0.5972	0.6175	0.6487	-
	Stacking Ensemble	0.6137	0.6214	0.6432	0.6663	-
	RF	0.6964	0.6811	0.7139	0.7100	-
	XGBoost	0.7320	0.7274	0.7548	0.7413	-
	Fine-tuned VGG19	0.7632	0.7119	0.8295	0.8444	-
	Fine-tuned Resnet50	0.7849	0.7988	0.8412	0.8785	-
Image–Text Multimodal Model	**BERT + Resnet50**	**0.8363**	**0.8536**	**0.8762**	**0.9218**	-

**Table 5:** The F-Score of the models on the test set. The best-performed model shows in bold.

Type	Models	#Nike	#Gucci	#CristianoRonaldo	#EdSheeran	All Hashtags
Metadata Classification	SVM	0.6867	0.7104	0.7191	0.7532	0.6864
	Stacking Ensemble	0.6550	0.7045	0.7265	0.7569	0.7208
	RF	0.7079	0.7103	0.7528	0.7697	0.7528
	XGBoost	0.7282	0.7564	0.7779	0.7638	0.7408
	Deep Dense layers	0.6987	0.6827	0.7211	0.7258	0.7169
Text Classification (Without OCR)	SVM	0.6395	0.6499	0.6993	0.6798	-
	Stacking Ensemble	0.6313	0.6238	0.6807	0.6968	-
	RF	0.5986	0.6704	0.6914	0.6835	-
	XGBoost	0.6250	0.6858	0.6851	0.7058	-
	Fine-tuned BERT	0.7460	0.7410	0.8155	0.8372	-
Text Classification (With OCR)	SVM	0.6493	0.6746	0.6858	0.6930	-
	Stacking Ensemble	0.6234	0.6432	0.6776	0.6625	-
	RF	0.6059	0.6807	0.6411	0.7167	-
	XGBoost	0.6955	0.6873	0.6798	0.7024	-
	Fine-tuned BERT	0.7547	0.7639	0.8317	0.8558	-
Image Classification	SVM	0.5275	0.5664	0.5721	0.5917	-
	Stacking Ensemble	0.5985	0.6067	0.6779	0.6776	-
	RF	0.6552	0.6775	0.7075	0.6922	-
	XGBoost	0.7082	0.7295	0.7633	0.7096	-
	Fine-tuned VGG19	0.7249	0.7198	0.8458	0.8513	-
	Fine-tuned Resnet50	0.7592	0.7743	0.8507	0.8627	-
Image–Text Multimodal Model	**BERT + Resnet50**	**0.8104**	**0.8359**	**0.8860**	**0.9106**	-

**Table 6:** Statistics of face clustering.

Hashtag	@CristianoRonaldo	@EdSheeran
Number of account images	2271	3095
Number of faces	8481	11,353
Number of clusters	65	24
Clusters with the highest number of members	1742 (20.54%)	1213 (12.30%)
Number of poor-quality faces	2778 (32.75%)	4206 (42.66%)
Number of faces in unknown clusters	1375 (16.21%)	5652 (57.33%)

## Data Availability

The dataset collected and analyzed in this study are publicly available in https://github.com/sajaddadgar/Multi-Modal-Deep-Learning-for-Detecting-Hashtag-Incongruity (accessed on 20 November 2022).

## Abbreviations

The following abbreviations are used in this manuscript:

AI	Artificial Intelligence
ML	Machine Learning
DL	Deep Learning
NLP	Natural Language Processing
CNN	Convolutional Neural Network
RNN	Recurrent Neural Network
LSTM	Long Short-Term Memory
GRU	Gated Recurrent Unit
SVM	Support Vector Machines
LR	Logistic Regression
DT	Decision Tree
NB	Naïve Bayes
RF	Random Forest
KNN	K-Nearest Neighbor
OCR	Optical Character Recognition
BERT	Bidirectional Encoder Representations from Transformers
ResNet	Residual neural network
MTCNN	Multi-task Cascaded Convolutional Networks
RFE	Recursive Feature Elimination
mAP	Mean Average Precision

## Appendix A

While conducting the aforementioned experiment using scikit-learn and TensorFlow, there were various hyperparameters that needed to be optimized before starting the training process. Hence, We tuned hyperparameters to find a set of optimal values that can maximize the performance of the models. As a tuning technique, we performed Grid Search on ML and DL models used in the classification module. Table A1 shows all hyperparameters and their optimal values used for building classifiers. In this table, the text classification models with and without are built with the same hyperparameters for better comparison. Moreover, we developed the multimodal model by fusing text and image classifiers with tuned hyperparameters. So we did not perform hyperparameter tuning for the image–text multimodal model. Moreover, the number of neurons and activation functions used for developing DL models is shown for each layer, respectively, in the optimal values. We also trained the stacking ensemble model with different ML algorithms for estimators and the final estimator and we selected the best combination.

(A1) NumberofFilters=(Numberofclasses+5)×3

Moreover, configuring the YOLO to train the custom object is required. Thus, we set the various hyperparameters, including the number of classes (i.e., number of clusters in celebrities, one class to detect brands’ logo), number of filters to detect three boxes per grid cell that have five variables consisting of classes, width, height, x, y, confidence rate (Formula (A1)).

**Table A1:** The hyperparameters used in the ML and DL models in the classification module and their optimal value obtained from the grid search technique.

Type	Models	Hyperparameters	Optimal Values
Metadata Classification	SVM	kernel = [linear, poly, rbf, sigmoid], C = [1, 10, 100, 1000]	kernel = rbf, C = 1
	Stacking Ensemble	estimators = [SVM, DT, XGBoost, NB, LR]	estimators = [SVM, DT, NB], final_estimator = LR
	RF	n_estimators = [10, 20, 50, 100, 200, 500], criterion = [gini, entropy, log_loss], max_depth = [None, 2, 5, 10], max_features = [sqrt, log2, None]	n_estimators = 10, criterion = gini, max_depth = None, max_features = sqrt
	XGBoost	loss = [log_loss, deviance, exponential], learning_rate = [0.01, 0.025, 0.05, 0.075, 0.1, 0.15, 0.2], max_depth = [3, 5, 8]	loss = log_loss, learning_rate = 0.15, max_depth = 3
	Deep Dense layers	Optimizer = [SGD, RMSprop, Adagrad, Adadelta, Adam, Adamax, Nadam], Learning rate = [0.0001, 0.001, 0.01, 0.1], Dropout = [0, 0.1, 0.2, 0.3, 0.4, 0.5, 0.6, 0.7, 0.8, 0.9], Number of neurons (hidden layers) = [16, 32, 64, 128, 256], Activation functions = [softmax, softplus, softsign, relu, tanh, sigmoid, hard_sigmoid, linear]	optimizer = Adamax, learning_rate = 0.1, Dropout = 0, number of neurons (hidden layers) = [32, 32, 32], Activation functions = [linear, relu, relu]
Text Classification	SVM	kernel = [linear, poly, rbf, sigmoid] C = [1, 10, 100, 1000]	kernel = rbf, C = 100
	Stacking Ensemble	estimators = [SVM, DT, XGBoost, NB, LR]	estimators = [SVM, XGBoost, NB], final_estimator = LR
	RF	n_estimators = [10, 20, 50, 100, 200, 500], criterion = [gini, entropy, log_loss], max_depth = [None, 2, 5, 10], max_features = [sqrt, log2, None]	n_estimators = 100, criterion = gini, max_depth = None, max_features = sqrt
	XGBoost	loss = [log_loss, deviance, exponential], learning_rate = [0.01, 0.025, 0.05, 0.075, 0.1, 0.15, 0.2], max_depth = [3, 5, 8]	loss = log_loss, learning_rate = 0.1, max_depth = 3
	Fine-tuned BERT	Optimizer = [SGD, RMSprop, Adagrad, Adadelta, Adam, Adamax, Nadam], Learning rate = [0.0001, 0.001, 0.01, 0.1], Dropout = [0, 0.1, 0.2, 0.3, 0.4, 0.5, 0.6, 0.7, 0.8, 0.9], number of neurons (hidden layers) = [16, 32, 64, 128, 256], Activation functions = [softmax, softplus, softsign, relu, tanh, sigmoid, hard_sigmoid, linear]	Optimizer = Adam, Learning rate = 0.001, Dropout = 0.1, number of neurons (hidden layers) = [256, 32], Activation functions = [relu, relu]
Image Classification	SVM	kernel = [linear, poly, rbf, sigmoid] C = [1, 10, 100, 1000]	kernel = rbf, C = 100
	Stacking Ensemble	estimators = [SVM, DT, XGBoost, NB, LR]	estimators = [SVM, XGBoost], final_estimator = LR
	RF	n_estimators = [10, 20, 50, 100, 200, 500], criterion = [gini, entropy, log_loss], max_depth = [None, 2, 5, 10], max_features = [sqrt, log2, None]	n_estimators = 100, criterion = gini, max_depth = None, max_features = sqrt
	XGBoost	loss = [log_loss, deviance, exponential], learning_rate = [0.01, 0.025, 0.05, 0.075, 0.1, 0.15, 0.2], max_depth = [3, 5, 8]	loss = deviance, learning_rate = 0.1, max_depth = 3
	Fine-tuned VGG19 and Resnet50	Optimizer = [SGD, RMSprop, Adagrad, Adadelta, Adam, Adamax, Nadam], Learning rate = [0.0001, 0.001, 0.01, 0.1], Dropout = [0, 0.1, 0.2, 0.3, 0.4, 0.5, 0.6, 0.7, 0.8, 0.9], number of neurons (hidden layers) = [16, 32, 64, 128, 256], Activation functions = [softmax, softplus, softsign, relu, tanh, sigmoid, hard_sigmoid, linear]	Optimizer = Adam, Learning rate = 0.001, Dropout = 0.2, number of neurons (hidden layers) = [256, 256, 16], Activation functions = [relu, relu, relu]
